# Tadalafil plus endothelin receptor antagonists in connective tissue disease-associated pulmonary arterial hypertension: A multicenter study on exercise capacity and cardiac outcomes

**DOI:** 10.1515/rir-2025-0012

**Published:** 2025-07-01

**Authors:** Qianwen Wu, Hua Ma, Dongyu Li, Huangshu Ye, Zhangdi Zhou, Ning Zhang, Yinsu Zhu, Ting Liu, Xiaoxuan Sun, Miaojia Zhang, Qiang Wang

**Affiliations:** Department of Rheumatology, The First Affiliated Hospital of Nanjing Medical University, Nanjing, Jiangsu Province, China; Department of Rheumatology and Immunology, The Affiliated Hospital of Xuzhou Medical University, Xuzhou, Jiangsu Province, China; Department of Rheumatology, The Affiliated Hospital of Yangzhou University, Yangzhou, Jiangsu Province, China; Department of Radiology, The Affiliated Cancer Hospital of Nanjing Medical University, Nanjing, Jiangsu Province, China; Department of Rheumatology, Wuxi People’s Hospital, Wuxi, Jiangsu Province, China

**Keywords:** pulmonary arterial hypertension, connective tissue disease, therapy, risk assessment, survival

## Abstract

**Background and Objectives:**

Pulmonary arterial hypertension (PAH) is a life-threatening condition that requires optimized medical therapy to maintain a low-risk profile. This study assessed the effects of initial PAH-specific combination therapy with tadalafil/sildenafil on clinical and functional outcomes in a real-world setting.

**Methods:**

We conducted a multicenter retrospective study of 85 patients diagnosed with connective tissue disease-associated PAH (CTD-PAH) *via* right heart catheterization from 2009 to 2023. Data on treatment regimens and efficacy measures, including 6-min walk distance (6MWD), N-terminal pro-B-type natriuretic peptide (NT-pro BNP), soluble suppression of tumorigenicity 2 (sST2), World Health Organization (WHO) functional class, risk stratification, treat-to-target status and survival, were collected.

**Results:**

Patients receiving initial combination therapy with endothelin receptor antagonists (ERAs) and phosphodiesterase-5 inhibitors showed varied improvements. The tadalafil plus ERAs combination significantly reduced NT-pro BNP levels and improved risk status (*P* < 0.05). Notable enhancements in 6MWD, soluble ST2, and WHO functional class were observed in the tadalafil plus ERA group (*P* < 0.001), but not in the sildenafil group (*P* > 0.05). Additionally, 1-year treat-to-target rates were higher in the tadalafil plus ERA group (73.5%) than in the sildenafil group (45.6%, *P* = 0.005).

**Conclusion:**

These findings suggest that tadalafil combined with ERAs leads to better improvements in exercise capacity, functional class, and treatment goals compared to sildenafil-based regimens, offering valuable insights for optimizing CTD-PAH treatment.

## Introduction

Pulmonary arterial hypertension (PAH) is a severe pulmonary vascular complication of connective tissue disease (CTD), and CTD-PAH is the second most common type after idiopathic PAH (IPAH).^[1]^ While therapeutic advancements have improved PAH outcomes, it remains a life-threatening disease.^[2]^

Approved drugs for PAH target the delicate balance between vasoconstriction and vasodilation.^[3,4]^ For newly diagnosed patients, initial combination therapy with an endothelin receptor antagonist (ERA) and a phosphodiesterase type-5 (PDE-5) inhibitor is recommended, following a risk-based assessment, mirroring the IPAH treatment algorithm.^[5]^ This combination therapy can effectively improve exercise capacity, cardiac biomarkers, functional class, and reduce hospitalizations.^[6, 7, 8, 9, 10, 11]^ Two commonly used PDE-5 inhibitors, sildenafil and tadalafil, have demonstrated positive effects in PAH patients.^[12, 13, 14, 15, 16]^ While the comparative effectiveness of initial tadalafil/sildenafil and ERA combination therapy remains understudied, real-world evidence suggests potential benefits for tadalafil plus an ERA, including easier administration and improved symptom and exercise capacity compared to sildenafil plus an ERA.

Therefore, this multicenter retrospective analysis aims to compare the real-world clinical outcomes (treat-to-target and survival) of these two initial combination therapy strategies in newly diagnosed CTD-PAH patients, with the ultimate goal of optimizing PAH treatment strategies.

## Methods

### Study Design

This was a multicenter retrospective analysis of data from consecutive newly diagnosed CTD-PAH patients admitted to the First Affiliated Hospital of Nanjing Medical University and three other participating tertiary medical centers (the Affiliated Cancer Hospital of Nanjing Medical University, Wuxi People’s Hospital and the Affiliated Hospital of Yangzhou University) between January 2009 and August 2023 (NCT05980728). It was approved by the Medical Ethics Committee of the First Affiliated Hospital of Nanjing Medical University (Number 2018-SR-333) and adhered to the principles outlined in the Declaration of Helsinki. All prospective participants had given written informed consent.

### Patients

Eligible patients included adults aged over 18 years with confirmed CTD and PAH. Patients were initiated on first-line oral combination therapy consisting of an ERA (bosentan, ambrisentan or macitentan) and a PDE-5 inhibitor (sildenafil or tadalafil).

**Diagnosis of CTDs:** Definite diagnosis of CTDs included Systemic Lupus Erythematosus (SLE) diagnosed according to the 2019 European Alliance of Associations for Rheumatology/American College of Rheumatology (EULAR/ACR) criteria, primary Sjogren’s Syndrome (pSS) defined according to the 2016 ACR criteria, Systematic Sclerosis (SSc) defined according to the 2013 ACR/EULAR criteria, Mixed CTD (MCTD) defined by Sharp criteria. Patients who met the classification criteria of two or more CTD at the same time were defined as having overlap syndrome (OS). Patients who had clinical and serological manifestations suggestive of systemic autoimmune diseases but did not fulfil the classification criteria for CTD were defined as undifferentiated CTD (UCTD).

**Diagnosis of PAH ^[5]^:** PAH was confirmed by right heart catheterization (RHC), with mean pulmonary arterial pressure (mPAP) > 20 mmHg, pulmonary arterial wedge pressure (PAWP) ≤15 mmHg, and pulmonary vascular resistance (PVR) > 2 Wood units at rest.

**Exclusion criteria:** (i) patients with IPAH and those with PAH co-induced by other factors;(ii) patients with significant evidence of restrictive or obstructive lung disease;(iii) patients who were lost to follow-up. The inclusion and exclusion criteria were detailed in supplementary material.

### Data Collection and Treatment Regimens

A standardized case report form was established to collect baseline and follow-up data, including demographic information, clinical characteristics, laboratory results, echocardiography parameters, right heart catheterization (RHC) parameters, and risk stratification.

**Baseline data:** (i) demographic data (age and gender), clinical characteristics [protopathic CTDs, course of PAH, WHO function class (WHO-FC) and 6-min walking distance (6MWD)], laboratory data, 2018WSPH risk stratification [WHO-FC, 6MWD, NT-pro BNP, right atrium pressure (RAP), cardiac index (CI) and mixed venous oxygen saturation (SvO_2_) were evaluated], the four-strata risk stratification [including WHO-FC, 6MWD and NT-pro BNP, and the Comparative, Prospective Registry of Newly Initiated Therapies for PH (COMPERA) 2.0 method was used to define the patient’s risk group], echocardiography (ECHO) parameters and RHC parameters; Follow-up data: According to the 2022 European Respiratory Society/European Society of Cardiology (ESC/ERS) guidelines, the four-strata risk stratification and ECHO parameters were performed at follow-up.

**Initial treatment regimens:** The choice of PAH-specific dual therapy was determined by the treating physician and encompassed six oral regimens, *i.e*., bosentan plus sildenafil, bosentan plus tadalafil, ambrisentan plus sildenafil, ambrisentan plus tadalafil, macitentan plus sildenafil and macitentan plus tadalafil. Dosing was: bosentan 62.5 mg twice daily (*b.i.d*.), increasing to 125 mg *b.i.d*. if needed; ambrisentan 5 mg once daily (*o.d*.), increasing to 10 mg *o.d*. if needed; macitentan 10 mg *o.d*.; sildenafil 20 mg three times daily (*t.i.d*.), increasing to 40 mg *t.i.d*. if needed; or tadalafil 20 mg *o.d*., increasing to 40 mg *o.d*. if needed. For patients classified as intermediate-high or high risk while on oral therapies, the addition of intravenous (*i.v*.) prostacyclin analogues or prostacyclin receptor agonist would be considered at the discretion of the treating physician. Alongside PAH-specific therapy, patients also received standard supportive care, including diuretics, oxygen therapy, and treatment for CTD (glucocorticoids and immunosuppressants).

**Sequential treatment:** Patients were eligible to switch from PDE-5 inhibitors to riociguat, or receive add-on therapy with prostacyclin analogues or prostacyclin receptor agonists if their condition deteriorated or they exhibited an inadequate response while on dual therapy. Further details regarding data collection, risk stratification, and treatment regimens can be found in the supplementary material.

### Endpoint and Follow-up

The primary endpoint of the study was defined as the duration from baseline to the first adjudicated treat-to-target status within 1 year, defined as simultaneously achieving:(i) WHO-FC I or II; and (ii) 6MWD > 440 m; and (iii) BNP < 50 ng/L or NT-pro BNP < 300 ng/L. The secondary endpoint of the study was defined as all-cause mortality.

Follow-up data were gathered through medical record reviews and telephone consultations, covering the period from the date of PAH diagnosis up to 1 year later. Extended follow-up was then conducted until January 30, 2024.

### Statistical Analysis

The statistical analyses were performed using SPSS version 26.0 (IBM Corp, Armonk, NY, USA), GraphPad Prism 9.0 (GraphPad Software, San Diego, USA), and R statistical software v4.3.2 (R Foundation for Statistical Computing, Vienna, Austria). Normality was assessed with the Kolmogorov-Smirnov test. Continuous variables were expressed as mean ± standard deviation (SD) or as medians with interquartile ranges. Categorical variables were expressed as absolute and relative frequencies (percentages). The comparisons of combined sildenafil and ERA versus tadalafil and ERA were analyzed by Student t-test or Wilcoxon test for baseline values, while chi-squared test or Fisher’s exact test was used to compare categorical variables. Changes in 6MWD, NT-pro BNP, soluble suppression of tumorigenicity 2 (sST2), WHO-FC and four-strata risk stratification between baseline and the first follow-up visit were assessed using the Student *t*-test or Wilcoxon test for paired groups, depending on the distribution of the variables. Cumulative treat-to-target rates and survival probabilities were evaluated through Kaplan-Meier analysis, with further comparisons conducted using the log-rank test. All statistical tests were two-sided, and a *P*-value of less than 0.05 was considered statistically significant.

## Results

### Demographics and Characteristics at Baseline

In this retrospective analysis, a total of 85 patients were included, reflecting the real-world nature of the study. The patient population was predominantly female, with nearly 96.5% of individuals being women, aligning with the higher prevalence of CTD-PAH among females. Within the cohort of patients with CTD-PAH, the most prevalent underlying CTD subgroups were SLE at 42.2%, followed by pSS at 27.1%, and SSc at 7.1%. The average age of the study participants was 43.64 years, with the majority (92.9%) falling into WHO-FC II-III and exhibiting significant hemodynamic impairment (Table 1).

**Table 1:** Demographics and characteristics at baseline

	All patients (*N* = 85)
Characteristics	
Age, years	43.64 ± 13.74
Female, *n* (%)	82 (96.5)
Protopathic CTDs	
SLE/pSS/SSc/MCTD/Overlap Syndrome/Other CTDs, *n*	36/23/6/5/6/9
Course of PAH, months	9.00 (3.00–35.50)
6MWD, m	419.95 ± 116.69
WHO-FC (I/II/III/IV), *n*	3/35/44/3
SLEDAI	6.00 (4.00–8.00)
Laboratory data	
eGFR, mL/min/1.73 m^2^	99.85 ± 24.15
NT-pro BNP, ng/L	1035.50 (353.55–2951.50)
sST2, μg/L	33.60 (23.10–55.38)
ESR, mm/h	29.00 (7.50–60.00)
CRP, mg/L	3.83 (2.53–12.50)
NLR	2.93 (1.66–4.87)
Risk stratification	
2018WSPH, Low/intermediate/high risk, *n*	29/30/26
4-strata risk Low/intermediate-low/ intermediate-high/ high risk, *n*	25/20/30/9
Right cardiac catheterization parameters	
Heart rate, beats/min	85.92 ± 12.09
mPAP, mmHg	45.75 ± 11.97
PVR, Wood	10.73 ± 6.89
RAP, mmHg	5.00 (3.00–8.00)
CI, L/min/m^2^	2.72 ± 0.89
Two-dimensional echocardiography parameters	
RADI, mm/m^2^	27.51 ± 4.46
RVDDI, mm/m^2^	28.02 ± 4.37
TAPSE/PASP, mm/mmHg	0.20 (0.16–0.30)
RV FAC, %	29.00 (24.00–34.00)
PASP	75.24 ± 20.73
PAH-targeted therapy	
Macitentan/Ambrisentan/ Bosentan, *n*	41/30/14
Triple therapy, *n* (%)	22 (25.9)
CTD therapy	
Glucocorticoids, *n* (%)	82 (96.5)
Immunosuppressants, *n* (%)	73 (85.9)

CTD: connective tissue disease, SLE: systemic lupus erythematosus, pSS: primary Sjogren’s syndrome, SSc: systematic sclerosis, MCTD: mixed connective tissue disease, PAH: pulmonary arterial hypertension, 6MWD: 6-minute walking distance, WHO-FC: WHO functional class, SLEDAI: systemic lupus erythematosus disease activity index, eGFR: estimated glomerular filtration rate, NT-pro BNP: N-terminal pro-B-type natriuretic peptide, sST2: soluble suppression of tumorigenicity 2, ESR: erythrocyte sedimentation rate, CRP: C-reactive protein, NLR: neutrophil/Lymphocyte ratio, mPAP: mean pulmonary artery pressure, PVR: pulmonary vascular resistance, RAP: right atrium pressure, CI: cardiac index, RADI: right atrial end-systolic diameter index, RVDDI: right ventricular end-diastolic basal dimension index, TAPSE/PASP: tricuspid annular plane systolic excursion/ pulmonary artery systolic pressure, RVFAC: right ventricular area change fraction, PASP: pulmonary artery systolic pressure.

The patients were stratified into two groups based on the type of PDE-5 inhibitors received: tadalafil group (*N* = 56) and sildenafil group (*N* = 29). There were no notable differences observed in exercise capacity, cardiac biomarkers, risk stratification, and hemodynamic characteristics between the tadalafil plus ERAs and sildenafil plus ERAs group at baseline, as outlined in detail in Table 2.

**Table 2:** Baseline characteristics of the grouping

	Tadalafil plus ERAs (*N* = 56)	Sildenafil plus ERAs (*N* = 29)	*P*-value
Characteristics			
Age, years	44.07 ± 13.44	42.79 ± 14.51	0.687
Female, *n* (%)	55 (98.2)	27 (93.1)	0.267
Protopathic CTDs			
SLE/pSS/SSc/MCTD/Overlap Syndrome/Other CTDs, *n*	23/17/5/3/4/4	13/6/1/2/2/5	0.653
Course of PAH, months	6.00 (3.00–33.50)	16.00 (2.50–63.00)	0.483
6MWD, m	428.98 ± 117.62	398.59 ± 114.28	0.309
WHO-FC (I/II/III/IV), *n*	1/24/29/2	2/11/15/1	0.831
SLEDAI	6.00 (4.00–10.00)	5.00 (4.00–7.50)	0.306
Laboratory data			
eGFR, mL/min/1.73 m^2^	98.67 ± 24.26	102.09 ± 24.21	0.541
NT-pro BNP, ng/L	1353.50 (466.40–3917.75)	948.55 (233.55–1825.25)	0.148
sST2, μg/L	33.41 (22.94–60.48)	34.34 (22.94–47.42)	0.658
ESR, mm/h	31.50 (4.75–71.50)	29.00 (14.00–56.00)	0.775
CRP, mg/L	3.76 (2.05–11.93)	4.02 (3.03–15.30)	0.363
NLR	3.22 (1.59–5.86)	2.51 (1.74–3.67)	0.376
Risk stratification			
2018WSPH, Low/intermediate/high risk, *n*	17/21/18	12/9/8	0.395
4-strata risk Low/intermediate-low/ intermediate-high/high risk, *n*	16/14/20/6	9/6/10/3	0.882
Right cardiac catheterization parameters			
Heart rate, beats/min	86.28 ± 11.81	85.16 ± 12.87	0.704
mPAP, mmHg	45.80 ± 11.34	45.66 ± 11.44	0.957
PVR, Wood	11.10 ± 7.34	10.01 ± 5.99	0.496
RAP, mmHg	5.00 (3.00–8.75)	5.00 (3.00–6.75)	0.609
CI, L/min/m^2^	2.69 ± 0.90	2.77 ± 0.88	0.710
Two-dimensional echocardiography parameters			
RADI, mm/m^2^	27.67 ± 4.57	27.20 ± 4.33	0.654
RVDDI, mm/m^2^	28.22 ± 4.66	27.63 ± 3.78	0.562
TAPSE/PASP, mm/mmHg	0.20 (0.16–0.33)	0.23 (0.18–0.28)	0.973
RV FAC, %	30.00 (24.00–34.00)	26.00 (22.50–36.18)	0.600
PASP	71.66 ± 20.49	82.14 ± 19.72	0.026
CTD therapy			
Glucocorticoids, *n* (%)	54 (96.4)	28 (96.6)	1.000
Immunosuppressants, *n* (%)	49 (87.5)	24 (82.8)	0.790

Continuous variables were expressed as mean ± SD or medians and interquartile ranges. Categorical variables were expressed as absolute and relative frequencies (percent). P-value for group comparisons. CTD: connective tissue disease, SLE: systemic lupus erythematosus, pSS: primary Sjogren’s syndrome, SSc: systematic sclerosis, MCTD: mixed connective tissue disease, PAH: pulmonary arterial hypertension, 6MWD: 6-minute walking distance, WHO-FC: WHO functional class, SLEDAI: systemic lupus erythematosus disease activity index, eGFR: estimated glomerular filtration rate, NT-pro BNP: N-terminal pro-B-type natriuretic peptide, sST2: soluble suppression of tumorigenicity 2, ESR: erythrocyte sedimentation rate, CRP: C-reactive protein, NLR: neutrophil/Lymphocyte ratio, mPAP: mean pulmonary artery pressure, PVR: pulmonary vascular resistance, RAP: right atrium pressure, CI: cardiac index, RADI: right atrial end-systolic diameter index, RVDDI: right ventricular end-diastolic basal dimension index, TAPSE/PASP: tricuspid annular plane systolic excursion/pulmonary artery systolic pressure, RVFAC: right ventricular area change fraction, PASP: pulmonary artery systolic pressure.

### Treatment

At baseline, all patients with CTD-PAH received combination therapy for a minimum of 3 months. Specifically, 34 patients were treated with tadalafil alongside macitentan, 21 with tadalafil plus ambrisentan, 1 with tadalafil plus bosentan, 7 with sildenafil in combination with macitentan, 9 with sildenafil plus ambrisentan, and 13 with sildenafil plus bosentan. Additionally, 22 patients (25.9%) were prescribed prostacyclin receptor agonist (selexipag) as an adjunct therapy as per the treating physician discretion. Notably, the majority of patients received glucocorticoids (96.5%) and immunosuppressants (85.9%) as part of their baseline etiological treatment.

During the first-year post-diagnosis, the majority of patients (87.0%) continued with their initial therapy, while 3.5% underwent treatment escalation and 9.5% experienced treatment reduction. Specifically, 3 patients (3.5%) received additional therapy with prostacyclin analogues or selexipag, 3 patients (3.5%) were switched from macitentan to ambrisentan, 1 patient (1.2%) was switched from ambrisentan to macitentan, 2 patients (2.4%) were de-escalated from triple therapy to dual therapy, and 6 patients (7.1%) shifted from dual therapy to monotherapy.

### Treatment Response at First Follow-up

**Figure 1:**
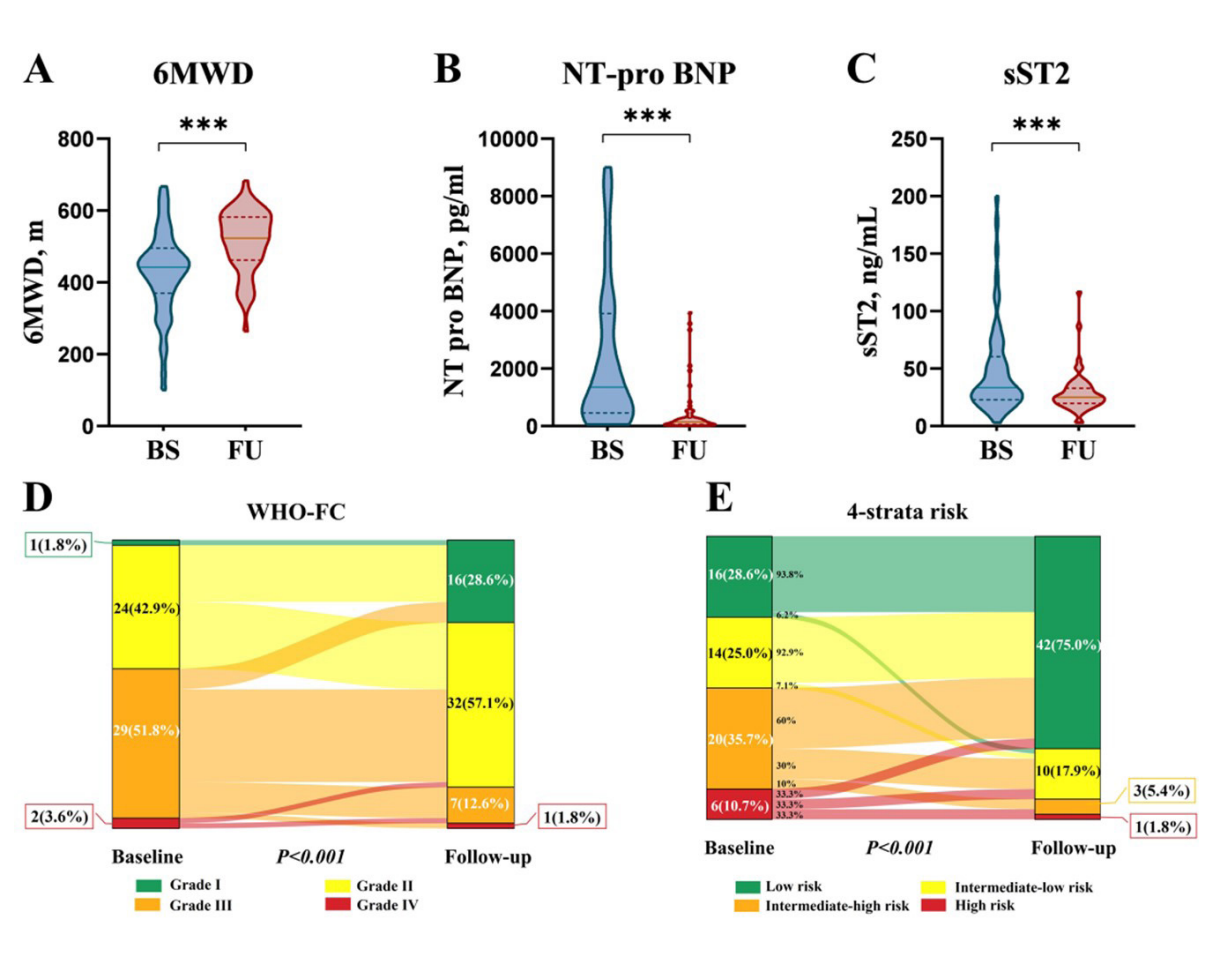
Effect of initial PAH-specific dual oral combination therapy with tadalafil plus ERAs on (A) 6-min walk distance (6MWD), (B) N-terminal pro-B-type natriuretic peptide (NT-pro BNP), (C) soluble suppression of tumorigenicity 2 (sST2), (D) World Health Organization functional class (WHO-FC), and (E) risk stratification at first follow-up visit in patients with CTD-PAH. ***P < 0.001.

The first follow-up assessment was conducted one year after PAH diagnosis. Initial combination therapy with tadalafil and ERAs significantly improved 6MWD, WHO-FC, NT-pro BNP and sST2 levels (*P* < 0.001; Figure 1 and Supplementary Table S1). Sildenafil plus ERAs decreased NT-pro BNP levels (*P* < 0.05), but showed no statistically significant difference in 6MWD, sST2 levels, and WHO-FC (*P* > 0.05, Supplementary Figure S1 and Supplementary Table S2).

Risk stratification differed between the tadalafil plus ERAs and sildenafil plus ERAs groups. At initial follow-up, the tadalafil plus ERAs group showed a higher proportion of low-risk patients (42/56, 75.0%) compared to the sildenafil plus ERAs group (16/27, 59.3%). Intermediate-low, intermediate-high, and high-risk classifications were distributed as 17.9%, 5.4%, and 1.8% in the tadalafil plus ERAs group, and 25.9%, 7.4%, and 7.4% in the sildenafil plus ERAs group, respectively (Figure 1 and Supplementary Figure S1). This difference was further accentuated after five years of extended follow-up, with 71.4% of the tadalafil plus ERAs group achieving low-risk stratification compared to 41.4% in the sildenafil plus ERAs group.

### Treat-to-target and Survival

**Figure 2:**
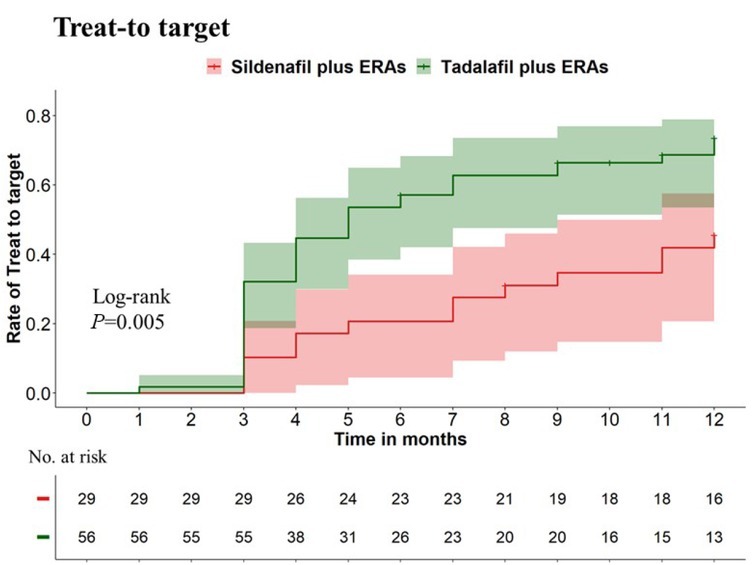
The cumulative rate of treat-to-target within the first year between patients receiving initial combination therapy of tadalafil and ERAs and those receiving sildenafil and ERAs. Individuals treated with tadalafil combined with ERAs were more likely and earlier to achieve treat-to-target than those treated with sildenafil plus ERAs, with 1-year treat-to-target cumulative rates of 73.5% vs. 45.6%, respectively (P = 0.005).

Patients treated with tadalafil combined with ERAs were more likely and earlier to achieve treat-to-target than those treated with sildenafil plus ERAs, with 1-year treat-to-target cumulative rates of 73.5% versus 45.6%, respectively (*P* = 0.005, Figure 2). In our extended follow-up period of more than five years, 92.5% of patients in the tadalafil plus ERAs group maintained treat-to-target, compared to 69.2% of patients in the sildenafil plus ERAs group.

The 1-, 3-, and 5-year survival rates for this cohort of CTD-PAH patients stood at 92.1%, 78.3%, and 78.3%, respectively, among those who received initial combination therapy (Supplementary Figure S2). Importantly, the 5-year survival probability was significantly elevated in patients who achieved treat-to-target compared to those who did not (93.3% *vs*. 59.7%, log-rank *P* < 0.001, Supplementary Figure S3).

### Safety and Tolerability

The use of PAH-specific dual therapy involving ERAs in combination with PDE-5 inhibitors was generally well tolerated, with adverse events consistent with the known side-effect profiles of the medications. In one case, ERA treatment was discontinued in a patient due to the development of anemia. Another patient had to be transitioned to dual therapy from triple therapy due to financial constraints, while three patients were switched to monotherapy because of poor compliance with the prescribed treatment regimen.

### Discussion

This real-world, multicenter observational study compared the effectiveness of initial combination therapy with tadalafil plus ERAs versus sildenafil plus ERAs in newly diagnosed CTD-PAH patients. Our results demonstrate that tadalafil plus ERAs led to earlier achievement of treat-to-target status. This was accompanied by significantly greater improvements in 6MWD, WHO-FC, and sST2 levels compared to the sildenafil plus ERAs group. While both treatment arms showed comparable improvements in NT-pro BNP and overall risk stratification, our findings suggest a potential advantage for tadalafil plus ERAs in achieving optimal early therapeutic responses.

These results underscore the importance of the 2022 ESC/ERS guidelines’ emphasis on a goal-oriented treatment strategy in PAH.^[2]^ The treat-to-target strategy, introduced in 2005 ^[17]^ and reinforced by the 2022 ESC/ERS guidelines, emphasizes achieving low-risk status for improved outcomes.^[2,18,19]^ Early detection, timely management, and transitioning patients out of intermediate or high-risk states swiftly are crucial for enhancing the prognosis of PAH.^[4]^ Achieving low-risk status is a critical determinant of long-term survival in PAH,^[20, 21, 22, 23]^ and our study reinforces the benefits of early and aggressive combination therapy to rapidly achieve this goal. The higher treat-to-target rate observed in the tadalafil plus ERAs group (73.5% *vs*. < 50% in the sildenafil plus ERAs group) suggests this combination may facilitate faster attainment of low-risk status and potentially improve long-term outcomes. This is further supported by our observation of significantly improved 5-year survival in patients who achieved treat-to-target status (93.3% *vs*. 59.7%).

Our findings contribute to the growing body of evidence supporting the superiority of combination therapy over monotherapy in CTD-PAH.^[21, 22, 23, 24, 25]^ While previous studies have demonstrated the benefits of initial combination therapy with an ERA and a PDE-5 inhibitor,^[2]^ our study provides valuable real-world data directly comparing the effectiveness of tadalafil and sildenafil in this context. A multitude of mechanisms mediated by PDE-5 inhibition have been documented,^[12, 13, 14, 15]^ including: (i) activation of the Nitric Oxide (NO)/cyclic Guanosine Monophosphate (cGMP)/Protein Kinase G (PKG) pathway, leading to decreased calcium influx through L-type calcium channels and increased calcium sequestration, resulting in vasorelaxation; (ii) suppression of DNA synthesis and cell proliferation, as well as induction of apoptosis in pulmonary artery smooth muscle cells (PASMCs), which play a role in the development of intimal hyperplasia and major vascular lesions in PAH; and (iii) augmentation of circulating endothelial progenitor cell numbers. The observed differences between the two PDE-5 inhibitors may be related to their distinct pharmacological profiles. While both drugs inhibit PDE-5 and enhance NO signaling, preclinical studies suggest tadalafil may offer additional benefits, including inhibiting hypoxic pulmonary vasoconstriction, attenuating hypoxia-induced tumor necrosis factor alpha (TNF-α) and interleukin (IL)-1β expression, and exhibiting more potent anti-proliferative and pro-apoptotic effects on pulmonary artery smooth muscle cells.^[26,27]^ These mechanisms may contribute to the improved clinical outcomes observed with tadalafil plus ERAs.

The higher proportion of patients receiving tadalafil plus ERAs (65.9%) compared to sildenafil plus ERAs (34.1%) in our cohort likely reflects real-world prescribing patterns, potentially influenced by tadalafil’s convenient once-daily dosing. The most commonly used combination therapy is tadalafil plus macitentan. The use of bosentan and sildenafil in a subset of patients (15.3%) reflects physician preferences and historical prescribing practices in China, where factors such as drug availability and familiarity may influence treatment choices. This imbalance in treatment allocation, while reflecting real-world practice, introduces a potential confounding factor that warrants consideration.

Our study has several limitations inherent to its observational design. The relatively small sample size, a consequence of the underutilization of combination therapy in routine clinical practice, limits the statistical power of our analysis. The non-randomized treatment allocation and potential influence of unmeasured confounders, including concomitant CTD medications, further complicate the interpretation of our findings. The specific impact of glucocorticoids and immunosuppressants on PAH symptoms in our cohort remains unknown and warrants further investigation.

Despite these limitations, our study provides valuable insights into the real-world effectiveness of initial combination therapy in CTD-PAH. The observed benefits of tadalafil plus ERAs over sildenafil plus ERAs warrant further investigation in larger, prospective studies designed to address the limitations of our observational analysis. Future research should focus on confirming these findings in diverse patient populations and exploring the potential mechanisms underlying the observed differences between tadalafil and sildenafil.

### Conclusion

Our study demonstrated that CTD-PAH patients treated with a combination of tadalafil and ERAs were more likely and earlier to achieve treat-to-target status than those receiving sildenafil plus ERAs. This treatment regimen was associated with significant enhancements in clinical and functional outcomes, providing robust evidence and valuable insights for optimizing the treatment of CTD-PAH. To further validate and refine these findings, larger-scale, multi-center studies incorporating rigorous methodologies and optimized treatment protocols are essential. Expectantly, multiple and novel therapeutic approaches are currently being evaluated in all phases of the drug development pipeline, some of which will hopefully provide meaningful improvements in outcomes for patients with PAH.

## Supplementary Information

*Supplementary materials are only available at the official site of the journal (www.rir-journal.com*).
